# The Vecta 46 intermediate catheter for mechanical thrombectomy of distal medium vessel occlusions: A single-center experience

**DOI:** 10.1177/15910199241283513

**Published:** 2024-10-14

**Authors:** Joo Won Choi, Yang Qiao, Tej I. Mehta, Thomas M. Clausen, Y. Jonathan Zhang, Samuel Tsappidi, Ferdinand K. Hui

**Affiliations:** 1 Department of Neurosciences, 21814 UC San Diego School of Medicine, La Jolla, CA, USA; 2 Department of Diagnostic and Interventional Imaging, 12340 The University of Texas Health Science Center at Houston, Houston, TX, USA; 3 Department of Interventional Radiology, 4002 The University of Texas MD Anderson Cancer, Houston, TX, USA; 4 Department of Radiology and Radiological Science, 1501 Johns Hopkins Medicine, Baltimore, MD, USA; 5 50677 John A. Burns School of Medicine, University of Hawai’i at Mānoa, Honolulu, HI, USA; 6 Department of Neurointerventional Surgery, 24796 The Queen's Medical Center, Honolulu, HI, USA; 7 Department of Neurosurgery, 24796 The Queen's Medical Center, Honolulu, HI, USA

**Keywords:** Stroke, distal medium vessel occlusion, aspiration, Vecta 46, thrombectomy

## Abstract

**Introduction:**

With emerging evidence supporting the clinical efficacy and safety of mechanical thrombectomy (MT) for distal medium vessel occlusions (DMVOs), MT devices specifically designed to navigate through smaller caliber and more delicate tortuous distal cerebrovasculature are required. This study describes our single-center experience using the AXS Vecta 46 intermediate catheter for first-line thromboaspiration of DMVOs.

**Methods:**

We identified all patients who underwent MT using the Vecta 46 for first-line thromboaspiration for primary or secondary DMVOs. We collected baseline clinical data, angiographic and clinical outcomes, as well as procedural complications. The primary outcome in question was the rate of successful recanalization, which was defined as a modified Thrombolysis in Cerebral Infarction score of ≥2b.

**Results:**

We identified 43 patients who underwent MT using the Vecta 46 catheter for thromboaspiration of 54 DMVOs. Intervened vessels included the M2 (23/54), M3 (19/54), and M4 (6/54) branches of the middle cerebral artery, A2 (1/54), A3 (1/54), and A4 (1/54) branches of the anterior cerebral artery, and P1 (1/54), P2 (1/54), and P4 (1/54) branches of the posterior cerebral artery. The median number of passes for primary DMVOs was 2 (IQR: 1–3) and 1 (IQR: 1–1.25) for secondary DMVOs. The rate of successful recanalization was 100% (18/18) for primary DMVOs and 80.6% (29/36) for secondary DMVOs. First-pass effect (FPE) was noted in 55.6% (30/54) of all primary and secondary DMVO cases. Improved short-term clinical outcomes were observed in both the primary (National Institute of Health Stroke Scale [NIHSS] shift: −5 [IQR: −14.25 to −0.25]) and secondary (NIHSS shift: −5 [IQR: −10 to −2]) DMVO groups. A total of six patients died during their hospitalization, though none were deemed procedural-related.

**Conclusions:**

Our study demonstrates the safety and efficacy of the Vecta 46 intermediate catheter for thromboaspiration of both primary and secondary DMVOs, achieving high rates of successful recanalization and FPE.

## Introduction

Advancements over the past decade have established endovascular mechanical thrombectomy (MT) as a cornerstone for treating acute ischemic stroke (AIS) in patients with large vessel occlusions (LVOs). With this came advancements in techniques and the development of more flexible, smaller devices capable of reaching more distal vessel occlusions. Emerging evidence points toward the potential clinical benefits of MT for both primary and secondary distal medium vessel occlusions (DMVOs) of the anterior and posterior circulations.^1–5^ Nevertheless, given the inherent risks of operating on smaller, more delicate distal occlusions with considerable angioarchitectural heterogeneity and the added technical challenges of navigating through tortuous vessels, further studies are required to identify the optimal instruments and techniques to utilize for MT of DMVOs.^6,7^

The AXS Vecta 46 intermediate catheter (Stryker Neurovascular, Fremont, CA, USA) is a 0.046-inch distal inner diameter catheter available in three lengths (125 cm, 146 cm, 160 cm) and is designed to access distal neurovasculature for support as well as aspiration purposes. Here, we conduct a retrospective, single-center investigation on the clinical safety, efficacy, and outcomes associated with the use of the Vecta 46 catheter for aspiration of both primary and secondary DMVOs in patients who underwent endovascular intervention for AIS. To our knowledge, this is the first study using the Vecta 46 catheter for thromboaspiration of DMVOs.

## Methods

This retrospective study was approved by the local institutional review board prior to its initiation. Between June 2023 and December 2023, we searched for all neurointerventional cases for which the Vecta 46 intermediate catheter was used. Within this cohort, we then identified patients with AIS who underwent MT using the Vecta 46 catheter for first-line thromboaspiration of both primary and secondary DMVOs, which included branches of the middle cerebral artery [M2–M4], anterior cerebral artery [A1–A4], and posterior cerebral artery [P1–P4].^6,7^ Baseline clinical data included age, sex, medical comorbidities, stroke etiology per the Trial of Org 10172 in Acute Stroke Treatment (TOAST) criteria, administration of intravenous thrombolysis prior to MT, and the NIH Stroke Scale (NIHSS) score at admission and discharge.^8^ We also collected the sites of occlusion, the need for rescue therapy with a stent retriever, time from groin puncture to recanalization, and time from last known well (LKW) to recanalization.

The primary outcome in question was the rate of successful recanalization, defined as a modified Thrombolysis in Cerebral Infarction (mTICI) score of ≥2b (>50% anterograde reperfusion to the ischemic territory of the target occlusion). Secondary outcomes included the NIHSS score shift from admission to discharge, the number of passes, and first-pass effect (FPE) which was defined as mTICI ≥ 2b after a single pass. Other secondary outcomes were the rates of procedural-related complications, including iatrogenic vessel dissection or perforation, periprocedural distal clot migration or reocclusion, vessel spasm, symptomatic and asymptomatic intracranial hemorrhage (ICH), and subarachnoid hemorrhage (SAH). All procedures were performed by three mid-career, experienced neurointerventionalists.

## Results

### Study cohort

At our institution, the Vecta 46 catheter was utilized in 136 total procedures. Among them, we identified 43 patients who underwent MT using the Vecta 46 for thromboaspiration of 54 total DMVOs. Our population had a mean age of 70.8 ± 13.8 years, with 27 (62.8%) male and 16 (37.2%) female patients. The median NIHSS score at presentation was 16 (IQR: 10–24). The mean age of patients with primary DMVOs (77.4 ± 10.3) was greater than those with secondary DMVOs (66.8 ± 14.2). Cardioembolism (58.1%) was the most common stroke etiology in both groups; 17 (39.5%) patients received intravenous thrombolysis with tenectaplase. Hypertension was the most common comorbidity (72.1%) across both groups. In the primary DMVO subgroup, atrial fibrillation was the next most common (62.5%) followed by hyperlipidemia (37.5%) and diabetes mellitus (25.0%). In the secondary DMVO subgroup, hyperlipidemia (51.9%) was the second most common comorbidity followed by hyperlipidemia (51.9%), and diabetes mellitus (29.6%). These results are shown in Table 1.

**Table 1.** Baseline patient characteristics stratified by DMVO type.

	Total (*n* = 43)	Primary (*n* = 16)	Secondary (*n* = 27)
Age^a^	70.8 ± 13.8	77.4 ± 10.3	66.8 ± 14.2
Gender			
Female	16 (37.2%)	8 (50%)	8 (29.6%)
Male	27 (62.8%)	8 (50%)	19 (70.4%)
Etiology			
Cryptogenic	10 (23.3%)	6 (37.5%)	4 (14.8%)
Cardioembolic	25 (58.1%)	9 (56.3%)	16 (59.3%)
Large artery	7 (16.3%)	–	–
Other (hypercoagulable state)	1 (2.33%)	1 (6.25%)	–
Small vessel	–	–	–
IV thrombolysis	17 (39.5%)	3 (18.8%)	14 (51.9%)
Comorbidities			
Hypertension	31 (72.1%)	12 (75%)	19 (70.4%)
Hyperlipidemia	20 (46.5%)	6 (37.5%)	14 (51.9%)
Diabetes mellitus	12 (27.9%)	4 (25%)	8 (29.6%)
Atrial fibrillation	17 (39.5%)	10 (62.5%)	7 (25.9%)
CAD	7 (16.3%)	–	7 (25.9%)
CHF	8 (18.6%)	3 (18.8%)	5 (18.5%)
Malignancy	7 (16.3%)	3 (18.8%)	4 (14.8%)
Previous stroke	7 (16.3%)	3 (18.8%)	4 (14.8%)
Tobacco use	3 (6.98%)	–	3 (11.1%)
Substance use	4 (9.3%)	1 (6.25%)	3 (11.1%)
Deep vein thrombosis	3 (6.98%)	1 (6.25%)	2 (7.41%)
Valvular disease	2 (4.65%)	–	2 (7.41%)
Groin-to-reperfusion^b^ (minutes)	39 (22–51)	44 (25–62)	36.5 (21.75–51.75)
LKW to Recanalization^b^ (minutes)	340 (223–750.5)	330 (221–740)	354.5 (215.5–958.75)
Initial NIHSS^b^	16 (10–24)	16.5 (10.25–20.75)	15 (8–26)
Discharge NIHSS^b^	8 (3–17)	6 (3.25–17.5)	8 (3–17)
NIHSS shift^b^	−5 (−11 to −1)	−5 (−14.75 to −0.25)	−5 (−10 to −2)
Complications			
In-hospital mortality	6 (14.0%)	2 (12.5%)	4 (14.8%)
sICH	3 (6.98%)	–	3 (11.1%)
aICH	1 (2.33%)	–	1 (3.7%)
aSAH	1 (2.33%)	1 (6.25%)	–
Clot migration	3 (6.98%)	1 (6.25%)	2 (7.41%)
Reocclusion	3 (6.98%)	2 (12.5%)	1 (3.7%)
Spasm	1 (2.33%)	–	1 (3.7%)

a Mean, standard deviation.

b Median, IQR.

aICH: asymptomatic intracerebral hemorrhage; aSAH: asymptomatic subarachnoid hemorrhage; CAD: coronary artery disease; CHF: chronic heart failure; IV: intravenous; LKW: last known well; NIHSS: National Institute of Health Stroke Scale; sICH: symptomatic intracerebral hemorrhage.

### Procedural outcome

Among the 54 DMVOs were 18 (33.3%) primary and 36 (66.7%) secondary DMVOs. Across both groups, the most common target vessel was the M2 (42.6%) followed by the M3 (35.2%). A final mTICI score of 2b, 2c, or 3 was achieved in all 100% (18/18) primary DMVOs and 80.6% (29/36) of secondary DMVOs. FPE was observed in 30 (55.6%) total cases, specifically in 7 (38.9%) primary DMVOs and 23 (63.9%) secondary DMVOs. 8 (44.4%) primary DMVOs and 2 (5.56%) secondary DMVOs required rescue therapy with a stent retriever (Trevo, Embotrap 3, ATLAS). The specific stent retriever device used for rescue therapy was provider dependent. The median number of passes for primary DMVOs was 2 (IQR: 1–3) and 1 (IQR: 1–1.25) for secondary DMVOs. Table 2 displays these findings.

**Table 2.** Procedural data across all DMVOs.

	Total (*n* = 54)	Primary (*n* = 18)	Secondary (*n* = 36)
Vessel			
M2	23 (42.6%)	10 (55.6%)	13 (36.1%)
M3	19 (35.19%)	6 (33.33%)	13 (36.1%)
M4	6 (11.1%)	–	6 (16.7%)
A1	–	–	–
A2	1 (1.85%)	–	1 (2.78%)
A3	1 (1.85%)	1 (5.56%)	
A4	1 (1.85%)	–	1 (2.78%)
P1	1 (1.85%)	–	1 (2.78%)
P2	1 (1.85%)	1 (5.56%)	–
P3	–	–	–
P4	1 (1.85%)	–	1 (2.78%)
Rescue therapy^a^	10 (16.7%)	8 (44.4%)	2 (5.56%)
Passes^a^	1 (1–2)	2 (1–3)	1 (1–1.25)
FPE^a^	30 (55.6%)	7 (38.9%)	23 (63.9%)
mTICI			
0	5 (9.26%)	–	5 (13.5%)
2A	2 (3.7%)	–	2 (5.41%)
2B	10 (18.5%)	2 (11.1%)	8 (21.6%)
2C	14 (25.93%)	4 (22.22%)	10 (27%)
3	23 (42.6%)	12 (66.7%)	11 (29.7%)
2B, 2C, 3	47 (87%)	18 (100%)	29 (80.6%)
2C, 3	37 (68.5%)	16 (88.9%)	21 (58.3%)

a Median, IQR.

FPE: first-pass effect; mTICI: modified thrombolysis in cerebral infarction.

Among both primary and secondary M2 occlusions, recanalization (mTICI 2b/2c/3) was achieved in 22 (95.7%) cases, 12 (52.3%) of which achieved FPE. Six (26.1%) M2 DMVOs required rescue therapy (Table 3). A similar recanalization rate was observed across all 18 (94.7%) M3 cases as well (Table 3). Other target vessels for which the Vecta 46 catheter achieved successful recanalization included the M4, A3, A4, P1, and P2 segments (Table 3). Recanalization was unsuccessful in one A2 occlusion and one P4 occlusion.

**Table 3.** Rates of successful recanalization, FPE, and rescue therapy by DMVO location.

	Successful Recanalization: mTICI 2B-3 (*n* = 47)	FPE (*n* = 30)	Rescue (*n* = 10)
Vessel			
M2	22 (95.7%)	12 (52.2%)	6 (26.1%)
M3	18 (94.7%)	14 (73.7%)	2 (10.5%)
M4	3 (50%)	1 (16.7%)	1 (16.7%)
A1	–	–	–
A2	–	–	–
A3	1 (100%)	1 (100%)	–
A4	1 (100%)	1 (100%)	–
P1	1 (100%)	1 (100%)	–
P2	1 (100%)	–	1 (100%)
P3	–	–	–
P4	–	–	–

FPE: first-pass effect; mTICI: modified thrombolysis in cerebral infarction.

### Clinical outcome

Median discharge NIHSS in the primary DMVO group was 6 (IQR: 3.25–17.5) and 8 (IQR: 3–17) in the secondary DMVO group (Table 1). A median NIHSS shift of −5 (IQR: −14.25 to −0.25) and −5 (IQR: −10 to −2) was observed in the primary and secondary DMVO groups, respectively (Table 1).

### Complications

A total of 6 (14.0%) patients died during their hospitalization (Tables 1 and 4). In four patients, this was due to a large core infarct from a proximal LVO leading to hemorrhagic transformation or malignant cerebral edema (Table 4). One patient died due to a new ischemic stroke that occurred post-procedurally contralateral to the intervened vessel likely from their underlying hypercoagulable state secondary to a malignancy. Another patient passed due to aspiration pneumonia. Symptomatic ICH (sICH) was observed in three patients, all in the secondary DMVO group (Table 1). One patient had asymptomatic ICH. Asymptomatic SAH (aSAH) manifested in 1 (2.33%) patient. Periprocedural clot migration was observed in 3 (6.98%) cases. Three (6.98%) patients experienced vessel reocclusion following initial MT and 1 (2.33%) patient had evidence of a nonflow limiting vasospasm on angiography. There were 0 instances of periprocedural iatrogenic vessel dissection or perforation. These findings are detailed in Table 1.

**Table 4.** Cause of in-hospital mortality across all patients.

Large infarction of proximal LVO (ICA/M1) leading to ICH and/or malignant cerebral edema	4
New ischemic stroke to contralateral vascular territory (hypercoagulable state, malignancy)	1
Other (aspiration pneumonia)	1

ICA: internal carotid artery; ICH: intracerebral hemorrhage; LVO: large vessel occlusion.

## Discussion

In this study, we demonstrate the safety and efficacy of the AXS Vecta 46 intermediate catheter for first-line aspiration of both primary and secondary DMVOs. Compared to previously reported rates of successful recanalization (72.4%) and FPE (52.4%) for aspiration of DMVOs in a recent review, we observed higher rates for both outcomes (87% and 55.6%, respectively) in our study.^6^ Of note, we observed a 100% (18/18) rate of successful reperfusion across all primary DMVOs, though compared to secondary DMVOs, a higher rate of rescue therapy (44.4%) and a lower rate of FPE (38.9%) were seen. Across both primary and secondary DMVOs, 10 (16.7%) cases required the use of rescue therapy with a stent retriever which was less frequent relative to previously cited numbers ranging from 31–41% in prior studies.^9,10^

Procedural observations with the Vecta 46 include its ease of trackability, ability to navigate proximal curves such as ophthalmic loops without the need for a microguidewire, as well as maintenance of luminal caliber without kinks. For distal branch access, an Aristotle 18 or 24 microguidewire is used to navigate to the target vessel branches, though in patients with challenging anatomy, a 21 microcatheter with a 0.014 microguidewire is preferred. Proximal support is obtained with a Sofia 6F aspiration catheter as an intermediary or directly from a guide catheter in select cases.

Our study contributes to the expanding body of literature investigating the use of new devices and techniques for MT of DMVOs.^11–13^ Despite multiple randomized control trials comparing the efficacy of first-line MT strategies (stent retriever, aspiration, combined), the applicability of these findings to DMVOs remains uncertain.^14,15^ DMVOs differ from LVOs in several respects: they are subject to higher vessel tortuosity, are less responsive to intravenous thrombolytics, and pose an increased risk of vessel injury such as iatrogenic dissection or perforation.^7^ Furthermore, most vessels intervened in these studies were M2 occlusions, which, given its heterogenous angioarchitecture, vary in vessel diameter and are thus less amenable to MT with traditional devices used for removal for LVOs.^6^ Given the current lack of dedicated, low-profile devices with smaller diameters—particularly aspiration catheters—specifically tailored for thrombectomy of DMVOs, use of such devices in future studies may yield improved clinical and procedural outcomes.^6^

While the data supporting MT of LVOs are far more extensive, there is a growing body of evidence highlighting the promising role of MT in the management of DMVOs. Recent investigations have demonstrated rates of early clinical improvement and functional independence (modified Rankin Scale [mRS] 0–2 at 90 days) following DMVO thrombectomy of the ACA and PCA territories—rates comparable to those observed with LVO thrombectomy.^1–3^ Caution must be taken however as the smaller volume of tissue supplied by such distal vessels raises the question of whether the potential therapeutic benefits of these techniques justify the risks associated with catheterization of thinner, more tortuous vessels.^7^

In our study, the Vecta 46 catheter demonstrated a favorable safety profile, with no cases of reported iatrogenic vessel damage, and similar rates of sICH, aSAH, and clot migration compared to prior studies.^6,9^ Cases in which the Vecta 46 catheter was unsuccessful were due to an inability to access the distal occlusion due to a tight vascular loop and insufficient catheter length limited by the patient's height. We observed an in-hospital mortality rate of 14%, similar to that reported in previous studies using aspiration catheters for MT of LVOs and/or DMVOs.^6,16^ As outlined in Table 4, none of the deaths were deemed procedure-related; they occurred in patients with large core infarcts or with extensive preexisting conditions.

There are several limitations to our study, including its retrospective, observational single-center design and lack of a control cohort. Another limitation was the absence of long-term functional outcomes (90-day mRS), although, in both primary and secondary DMVO groups, we observed improved early clinical outcomes as reflected by the NIHSS shift (Table 1).

## Conclusion

Our initial experience using the AXS Vecta 46 intermediate catheter demonstrates its clinical safety and efficacy for thromboaspiration of primary and secondary DMVOs, with higher rates of successful recanalization and FPE relative to prior reported rates. Given the considerable clinical and architectural variability observed with distal cerebrovascular occlusions, larger studies with more diverse vessel heterogeneity are needed to determine the optimal candidates and first-line strategies for thrombectomy of DMVOs.
